# The landscape of cardiovascular care in pediatric cancer patients and survivors: a survey by the ACC Pediatric Cardio-Oncology Work Group

**DOI:** 10.1186/s40959-019-0051-8

**Published:** 2019-10-23

**Authors:** Thomas D. Ryan, William L. Border, Carissa Baker-Smith, Ana Barac, Matthew J. Bock, Mary M. Canobbio, Nadine F. Choueiter, Devyani Chowdhury, Katheryn E. Gambetta, Julie S. Glickstein, Lavanya Kondapalli, Seema Mital, Vasum Peiris, Russell J. Schiff, Robert L. Spicer, Jeffrey A. Towbin, Ming Hui Chen

**Affiliations:** 1 Heart Institute, Cincinnati Children’s Hospital, University of Cincinnati College of Medicine, 3333 Burnet Ave, MLC 2003, Cincinnati, OH 45229 USA; 2 Children’s Sibley Heart Center, Children’s Healthcare of Atlanta, Atlanta, GA USA; 3 Department of Pediatrics, University of Maryland School of Medicine, Baltimore, MD USA; 4 MedStar Heart and Vascular Institute, Georgetown University, Washington, DC, USA; 5 Loma Linda University Children’s Hospital, Loma Linda, CA USA; 6 Ahmanson/UCLA ACHD Center, UCLA Health, Los Angeles, CA USA; 7 The Children’s Hospital at Montefiore, Albert Einstein College of Medicine, Bronx, NY USA; 8 Cardiology Care for Children, Lancaster, PA USA; 9 Ann and Robert H Lurie Children’s Hospital of Chicago, Chicago, IL USA; 10 Morgan Stanley Children’s Hospital of New York, Columbia University Vagelos College of Physicians and Surgeons, New York, NY USA; 11 UC Health Heart & Vascular Center – Anschutz, University of Colorado School of Medicine, Aurora, CO USA; 12 The Hospital for Sick Children, University of Toronto, Toronto, Canada; 13 Department of Health and Human Services, Food and Drug Administration Center for Devices and Radiological Health, Children’s National Health System and the George Washington University School of Medicine and Health Sciences, Washington, DC, USA; 14 Cohen Children’s Medical Center of New York – Northwell Health, New Hyde Park and Huntington, NY USA; 15 Children’s Hospital and Medical Center, University of Nebraska Medical Center, Omaha, NE USA; 16 Le Bonheur Children’s Hospital, The University of Tennessee Health Science Center, St. Jude Children’s Research Hospital, Memphis, TN USA; 17 Departments of Cardiology and Pediatrics, Boston Children’s Hospital, Dana-Farber Cancer Institute/Harvard Cancer Center, and Harvard Medical School, 300 Longwood Ave, Boston, MA 02115 USA

**Keywords:** Pediatrics, Cardio-oncology, Cancer, Survey, ACC, Cardiology, Cardiac risk factors, Echocardiography

## Abstract

**Objective:**

To enhance the understanding of cardiovascular care delivery in childhood cancer patients and survivors.

**Study design:**

A 20-question survey was created by the Pediatric Cardio-oncology Work Group of the American College of Cardiology (ACC) Cardio-oncology Section to assess the care, management, and surveillance tools utilized to manage pediatric/young adult cardio-oncology patients. The survey distribution was a collaborative effort between Cardio-oncology Section and membership of the Adult Congenital and Pediatric Cardiology Section (ACPC) of the ACC.

**Results:**

Sixty-five individuals, all self-identified as physicians, responded to the survey. Most respondents (*n* = 58,89%) indicated childhood cancer patients are regularly screened prior to and during cancer therapy at their centers, predominantly by electrocardiogram (75%), standard echocardiogram (58%) and advanced echocardiogram (50%) (i.e. strain, stress echo). Evaluation by a cardiologist prior to/during therapy was reported by only 8(12%) respondents, as compared to post-therapy which was reported by 28 (43%, *p* < 0.01). The most common indications for referral to cardiology at pediatric centers were abnormal test results (*n* = 31,48%) and history of chemotherapy exposure (*n* = 27,42%). Of note, during post-treatment counseling, common cardiovascular risk-factors like blood pressure (31,48%), lipid control (22,34%), obesity & smoking (30,46%) and diet/exercise/weight loss (30,46%) were addressed by fewer respondents than was LV function (72%).

**Conclusions:**

The survey data demonstrates that pediatric cancer patients are being screened by EKG and/or imaging prior to/during therapy at most centers. Our data, however, highlight the potential for greater involvement of a cardiovascular specialist for pre-treatment evaluation process, and for more systematic cardiac risk factor counseling in posttreatment cancer survivors.

## Introduction

Every year in the United States, cancer is diagnosed in more than 15,700 patients under 20-years of age [1]. Improved diagnosis and treatment have led to a dramatic increase in survival, which now exceeds 80% at 5-years after diagnosis, and translates to approximately 450,000 individuals living with a history of childhood cancer [2]. This improved survival is accompanied by an increased rate of associated long-term cardiovascular complications, which is the leading cause of both morbidity and mortality in long-term childhood cancer survivors [3, 4]. Despite the increasing awareness of cardiovascular morbidity in these patients, limited information exists on cardiovascular care delivery to this pediatric population. Therefore, members of the American College of Cardiology (ACC) Cardio-oncology Section and the ACC Pediatric Cardio-oncology Work Group, representing 15 different pediatric cardiology centers performed a practice survey of cardiology providers in the US caring for pediatric cancer patients. The purpose of this survey was to improve understanding of what groups of young childhood cancer survivors are being followed by pediatric cardiologists and for which indications, and what guidelines, if any, cardiologists use in their evaluation, management, and follow-up of cancer survivors.

## Method

In November 2017, a 20-question survey created by the multi-center Pediatric Work Group of the ACC Cardio-oncology Section was distributed to the members of the Adult Congenital and Pediatric Cardiology Section (ACPC) of the ACC asking “any practitioner who cares for [pediatric and/or adolescent cardio-oncology] patients” to answer “who is managing care, what methods of surveillance are employed, and how treatment is carried out.” The survey questionnaire is attached in the Additional file 1. Survivors were defined as those patients who have already undergone cancer therapy. Of note, patient demographics were not available for this study. Results are presented as count (%). Chi-square or the Fisher-Exact test was used for testing relationships between categorical variables where appropriate. Data analysis was performed on IBM SPSS version 24 (IBM Corp. Released 2015. IBM SPSS Statistics for Mac OS, Version 24.0. Armonk, NY: IBM Corp.). For some questions, not all participants replied, and this is indicated in the text.

## Results

A total of 65 individuals, all self-identified as physicians, responded to the survey. Of the respondents, 46 (71%) were affiliated with an academic institution, 11 (17%) in a practice owned by a hospital or larger institution, and the remainder owned by a corporation (*n* = 4, 6%), either privately owned (*n* = 3, 5%), or other (*n* = 1, 2%). The majority, 34 (52%), of those who returned the survey worked in a setting that had a cancer survivorship clinic, with 12 (18%) working in an environment where cardiology was embedded in the cancer survivorship clinic.

### Which patients are seen, and by whom?

**Fig. 1:**
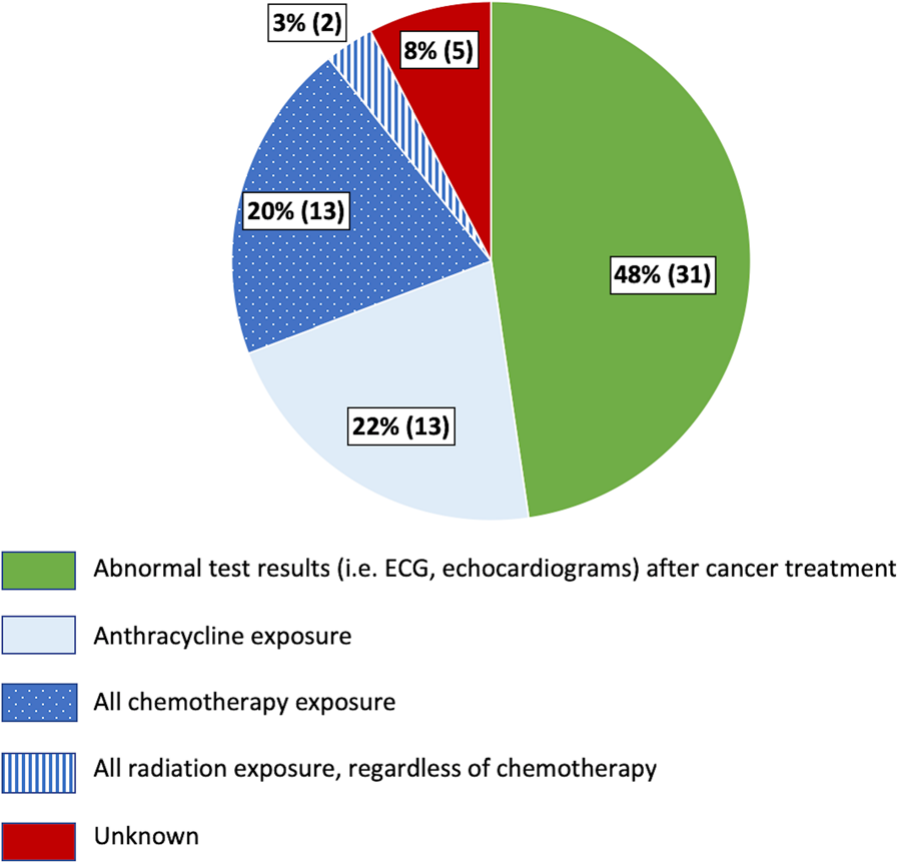
Reasons for referral to a cardiologist in childhood cancer patients undergoing cancer care. Responses to the question “Which of the following patient populations are referred for assessment by a cardiologist at your institution?” *N* = 65. Abnormal testing result is the major reason for cardiology referral

When asked “Who is responsible for managing cardio-oncology issues”, 31 (48%) respondents indicated that cardio-oncology care was shared between Pediatric Cardiology and Pediatric oncology, while 26 (40%) replied Pediatric Cardiology only and 2 (3%) Oncology only. Interestingly, only 3 (5%) respondents worked at an institution with a dedicated cardio-oncology service; the remainder listed “unknown” or “other”. When asked “Which of the following patients are seen for cardio-oncology at your institution”, 31 (48%) respondents indicated that their institution followed pediatric cancer survivors only, as opposed to 23 (35%) who follow both pediatric and adult cancer survivors; 11 (17%) marked “unknown” or “does not apply”. Regarding indications for referral of patients with cancer to a cardiologist, 31 (48%) responded those patients with abnormal test results [i.e. electrocardiogram (ECG), echocardiogram], 14 (22%) responded those treated with anthracyclines, 13 (20%) responded those treated with any type of chemotherapy, and 2 (3%) responded those with treated with radiation therapy (Fig. 1). Once patients had undergone therapy (i.e. survivors), the majority (*n* = 36, 55%) had at least one visit with a cardiologist, if there was an abnormality found on Children’s Oncology Group (COG) recommended testing. One-quarter of respondents indicated that patients were seen by a cardiologist at all COG recommended surveillance intervals (*n* = 15, 23%).

### Cardiac surveillance methods, pre-treatment and post-treatment

A majority of respondents (*n* = 58, 89%) were in a practice environment where patients receive baseline cardiac screening prior to and during cancer therapy. Since this was a survey directed at physicians’ practice behavior, no further information on demographics of patients who underwent screening were available. Most of the respondents reported that cancer survivors were assessed by ECG (75%), standard echocardiographic modalities (58%), and advanced echocardiographic modalities such as strain imaging, or stress echocardiography (50%) (Table 1). For patients who were undergoing active therapy, respondents reported that evaluation intervals were determined based on COG recommendations for cardiac screening in cancer patients (*n* = 28, 43%) or chemotherapy-specific protocols (*n* = 18, 28%); 14 (22%) respondents did not know the recommended screening interval for pediatric cancer survivors. Once patients were identified as cancer survivors, the reported usage of ECG and echocardiogram by cardiologists was similar to that reported for prior to cancer therapy (ECG: 75% vs 66%, *p* = 0.27; standard echocardiogram: 58% vs 55%, *p* = 0.77 and advanced echocardiogram: 50% vs 54%, *p* = 0.66). However, 12% of cardiologists report that patients were evaluated by a cardiologist prior to or during cancer therapy, whereas 43% of cardiologists report that patients are seen post-treatment (*p* < 0.01). Similarly, cardiologists reported use of cardiac MRI increased post-treatment (3% vs.17%, *p* < 0.01) (Table 1).

**Table 1:** Comparison of cardiology evaluation and cardiovascular imaging modalities in patients before/during treatment versus during survivorship care, by provider report. Patients were more likely to be evaluated by a cardiologist post-treatment or during survivorship care than prior/during cancer treatment. There was an increase in use of Cardiac MR imaging as part of survivorship care

Cardiac Visit/Cardiac Testing	Before/During Treatment (*N* = 64)	After Treatment/Survivorship (*N* = 65)	*P*-value
Evaluation by a Cardiologist	8 (12%)	28 (43%)	< 0.01
Electrocardiogram	48 (75%)	43 (66%)	0.27
Extended arrhythmia monitoring	2 (3%)	7 (11%)	0.16
Echocardiogram, standard^a^	37 (58%)	36 (55%)	0.77
Echocardiogram, advanced^b^	32 (50%)	35 (54%)	0.66
Cardiac magnetic resonance imaging	2 (3%)	11 (17%)	< 0.01
Radionuclide imaging (e.g. MUGA)	0 (0%)	2 (3%)	0.50
Serum biomarkers	5 (8%)	n/a	–
Cardiopulmonary exercise testing	n/a	5 (8%)	–
None	0 (0%)	0 (0%)	1.00
Other/Unknown/not applicable	9 (14%)	8 (12%)	0.76

*MUGA* Multi-gated acquisition scan, *n/a* Answer was not an option for this question

^a^Including shortening fraction and/or ejection fraction; ^b^including diastolic parameters, strain imaging, and stress echocardiography

### Ventricular dysfunction and cardiac therapies

**Fig. 2:**
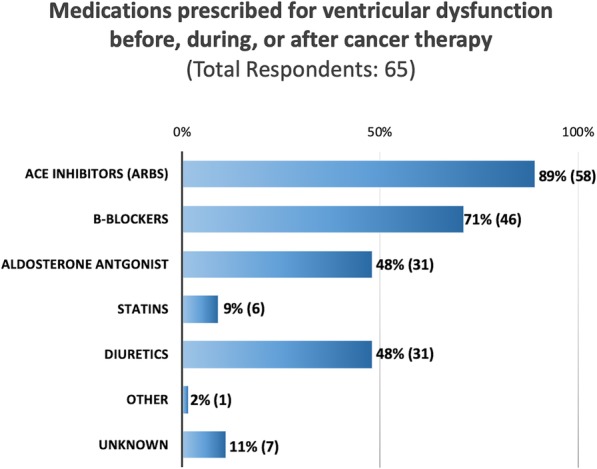
Medications prescribed for patients experiencing ventricular dysfunction before, during, or after cancer therapy, as reported by providers at pediatric centers (*N* = 65). ACE = angiotensin converting enzyme; MRB = mineralocorticoid receptor blocker. Unknown = provider answered they did not know

The decision to start treatment for ventricular dysfunction was based on left ventricular shortening fraction in 25% of respondents (*n* = 16), left ventricular ejection fraction in 28% of respondents (*n* = 18), strain values in 3% (*n* = 2), or abnormal values in any of the prior three measurements in 34% of respondents (*n* = 22). Six (9%) respondents answered “other” or “unknown”. Provider responses for types of therapies used included angiotensin converting enzyme (ACE) inhibitors, beta blockers, mineralocorticoid receptor blockers, statins, and diuretics (Fig. 2).

### Cardiovascular risk factor management

**Fig. 3:**
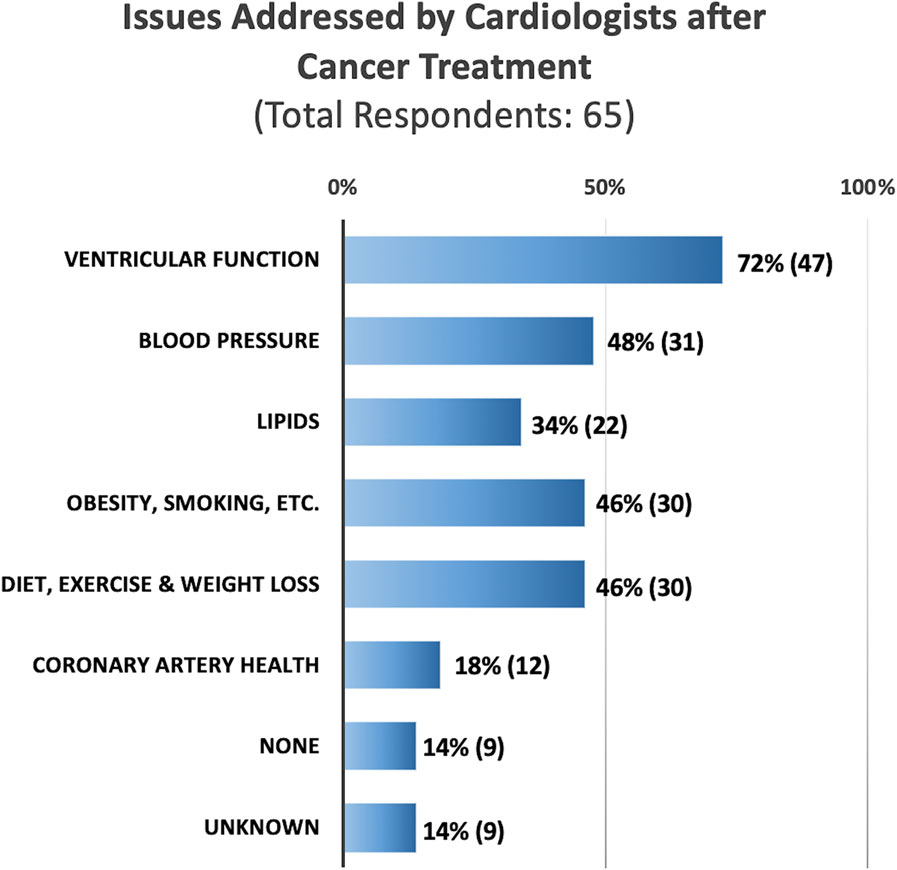
Cardiovascular risk factors discussed with patients after cancer therapy (survivors), as reported by pediatric providers (*N* = 65). CV = cardiovascular. Unknown = respondent did not know the answer

More than half (*n* = 37, 57%) indicated that a cardiologist was not involved in pre-treatment decisions of whether to use cardioprotective agents, such as dexrazoxane in combination with anthracycline. Ventricular dysfunction was the most common cardiovascular issue discussed with cancer survivors by cardiologists (*n* = 47, 72%) during their visit. In terms of cardiovascular risk factors, respondents also addressed blood pressure management (*n* = 31, 48%), diet and exercise (*n* = 30, 46%), other cardiovascular risk factors (smoking, obesity, etc.; *n* = 30, 46%), lipids (*n* = 22, 34%), and coronary artery health (*n* = 12, 18%) with survivors; 9 (14%) reported addressing none of these issues (Fig. 3), suggesting that the majority did discuss at least one modifiable cardiac risk factor with their patients. Additionally, 18 (28%) reported giving specific exercise prescription to survivors while 9 (14%) reported no discussion of exercise took place and 30 (46%) marked it as unknown. Importantly, of the modifiable cardiac risk factors queried, no one single cardiac risk factor was discussed by more than 50% of the respondents (Fig. 3). Therefore, while LV dysfunction is widely discussed, there is room to improve the emphasis on addressing all the traditional modifiable cardiac risk factors when counseling survivors.

### Other cardiovascular morbidities and advanced therapies

More than half of the survey respondents were at centers that offered heart transplantation (*n* = 41, 63%) and ventricular assist device (VAD; *n* = 41, 63%). One-third of respondents (21/63, 33%) did not know the minimum time off-therapy or in remission before a patient could be considered for heart transplant, while 8 (13%) reported a minimum time of at least a year, 8 (13%) reported at least 2 years and 6 (10%) reported greater than 5 years after cancer treatment. Similarly, 44% of providers (27/61) did not know the minimum time off-therapy or in remission before a patient could qualify for a VAD, suggesting a lack of uniform standards in the consideration of pediatric cancer survivors for advanced heart failure therapies.

## Discussion

In this study, we found variation in how pediatric patients treated with cardiotoxic chemotherapy were followed, both during treatment and post-exposure. Nearly 90% of survey respondents indicated cancer patients were screened prior to and during therapy, primarily with ECG and echocardiogram; only 12% reported evaluation by a cardiologist prior to therapy. According to the survey, only 20% of survivors were seen in a survivor clinic with cardiology involvement while 50% were seen in a survivor clinic without cardiology involvement. The data suggest an opportunity for cardiologists to partner with oncology teams at critical periods in disease management. We believe that a low threshold for early cardiology involvement in identifying and managing high risk patients may be considered. Even in this survey that is biased towards tertiary care centers with advanced HF therapies (63% respondents), our data suggests opportunity to improve survivorship care. Aside from addressing ventricular dysfunction, data from this study suggest an opportunity to increase efforts in cardiovascular risk factor management when seeing pediatric cancer survivors.

### Developing standardized practice

We observed variability in the overall cardiac care delivery to this population. Potential reasons for the variability can range from provider knowledge, referral practices, tailored care for a diverse population with differing anti-cancer exposures, and/or the need for more standardized cardiology protocols in this new field. A number of best practice and consensus statements have been published regarding the diagnosis, prevention, and management of cardiovascular complications of cancer therapies in adults [3–12]. With the exception of adult survivors of childhood cancers, there is a dearth of evidence-based literature that can guide screening and management of cardiac disease in children and adolescents actively undergoing therapy and in those who survived cancer treatment but yet to have reached adulthood. In 1992, Steinherz and colleagues published the first imaging recommendations in children [13]. The COG published its first long-term survivor follow-up guidelines based on expert opinion in 2003, with serial updates every several years, providing specific follow-up recommendations based on age at chemotherapy treatment, thoracic radiation exposure, and cumulative anthracycline dose (www.survivorshipguidelines.org). The 2013 American Heart Association (AHA) Scientific Statement provides a detailed review of the literature on the subject, identifies areas of future research, and provides some guidance regarding evaluation and management of survivors of pediatric and adolescent cancers [14]. All these publications highlight the relative lack of and need for more evidence-based data to assist recommendations for monitoring, prevention, and treatment guidelines [14]. Additionally, the International Late Effects of Childhood Cancer Guideline Harmonization Group publication, a large meta-analysis of multiple international consensus based recommendations on the topic, highlighted the need for long-term follow-up studies assessing the efficacy of screening on outcome measures or cost effectiveness [15].

In the case of pediatric and adolescent patients, guidelines have been produced to address management of heart failure in general but are not specific to cardio-oncology [16]. While the COG does have guidance for following cancer survivors, there is a dearth of guidelines for monitoring patients during active cancer therapy, or after cardiac abnormalities develop. This gap in knowledge of how to manage a large proportion of pediatric cancer patients needs to be addressed. For example, while multiple clinical trials in adults have reported a benefit with ACE inhibitors [17], there are very few pediatric trials in cancer patients with mixed results. A recent trial that randomized 84 pediatric patients with leukemia to receive 6 months of ACE inhibitor did not show a difference in left ventricular systolic dysfunction between treatment and placebo groups; however, fewer patients in the treatment group showed an increase in pro-brain natriuretic peptide compared to the placebo group [18]. Moreover, some authors have raised concern that use of ACE inhibitors in pediatric patients could possibly exacerbate a unique pathologic remodeling pattern [19]. Fortunately, current multi-center studies are recruiting participants to better understand the development of cardiotoxicity during therapy and in survivors [20], and the utility of prophylactic medical therapy to prevent development of ventricular dysfunction [21].

The current survey showed a variety of tools being used to assess ventricular function (Table 1). Adult-based guidelines support the use of many of these methods, but stress the importance of using the same technique throughout care for a given patient [8]. While patients are being primarily followed by oncologists for their cancer care, the primary specialty responsible for the long-term medical sequelae of cancer therapy and maintenance of health remains to be defined [22]. In the future, the timely early inclusion of cardiovascular specialists into the care of patients with cancer may improve outcomes. Cardiologists can help optimize cardiac risk factor management and more importantly, the primary prevention of cardiovascular events. Collaboration of cardiologists and oncologists in clinical practice and also research will continue to make gains on the survival of childhood cancer patients. Further areas of research would include, but not be limited to: cardioprotective therapy, appropriate cost-effective screening tools; role of incorporation of advanced imaging techniques into surveillance, use of biomarkers, anticipatory guidance and preventive care; and appropriate therapeutic strategies from medical therapy to advanced cardiac support.

### Prevention and education

A 2015 survey of Cardiology program directors predominately at adult centers, carried out by the ACC Cardio-oncology Section, found that > 70% felt that the implications of cancer treatment on cardiovascular health were an important consideration in patient care, and yet almost 40% of the participants did not feel confident in dealing with such issues or gave themselves an average rating when asked about their understanding of pertinent issues [23]. A more recent 2019 ACC survey of cardiology program directors showed that only 9% of programs had Cardio-oncology-specific training opportunities, and all of them require prior cardiology fellowship training [24]. Furthermore, there was “no formalized training for pediatric cardio-oncology” in the United States [23]. To improve the educational gaps in cardio-oncology for practicing physicians practice, “a number of live courses have been developed” over the last several years, and “there ..[has been] a greater number of cardio-oncology–focused sessions [included] at national meetings” [24]. However, the most effective way to disseminate standardized education and practice guidelines of cardio-oncology among new physicians is to incorporate it as part of Cardiology fellowship curriculum [25]. As being done in adult cardiology programs, systematic integration of cardio-oncology into fellowship training would likely improve the knowledge base, and practice among new pediatric cardiology physicians.

While there is an understanding of the importance of cardiovascular care for pediatric patients during and after cancer therapy, there is a lack of operationalized care and opportunities for improvement. In pediatric centers, care for the cardio-oncology patient often has resided in highly specialized heart failure clinics. However, studies consistently show that the cumulative burden of chronic cardiovascular conditions is substantial in childhood cancer survivors as they age, and there is a need to develop a more comprehensive, holistic and accessible approach to patient care [26]. The involvement of general cardiologists is vital to such an approach. Importantly, recent studies report that traditional risk factors such as hypertension, diabetes, obesity and smoking can further potentiate treatment-associated late effects [27, 28], and is an important point for medical intervention. For example, long-term HL survivors treated in childhood with radiation therapy had significantly higher risk of obstructive CAD and valve disease with higher blood pressures [26]. Involvement of general cardiologists and primary care providers, therefore, seems to be important in the cardiac care of this population as they grow and age.

### Limitations and future directions

This study is limited in being a voluntary sample of selected individuals, i.e. those involved in the ACC Adult Congenital and Pediatric Cardiology Section. Another limitation is the lack of detailed demographics on respondents. Therefore, indications for imaging could not be assessed.

There is a need for more multi-institutional studies in pediatric cancer survivors along with research examining the specific mechanisms of late cardiovascular effects, for which little research exists. Biological studies are also important in formulating suitable interventions that may have longer lasting effects. Intervention studies such as methods of cardiovascular risk factor modification and use of cardioprotective agents to mitigate the risk of late effects are also vital.

## Conclusions

The study survey data showed that pediatric cancer patients are being regularly screened by EKG or imaging prior to/during therapy at most centers. Despite some limitations, we did identify a potential area of intervention by cardiologists in terms of cardiac risk factor modification for pediatric cancer survivors. Future studies are also needed to explore the reasons for observed differences in practice, and to help guide future efforts by cardiologists in the rapidly developing field of pediatric cardio-oncology.

## Supplementary information

**Additional file 1.** Questions for Pediatric Cardio-Oncology Practice Survey.

## Data Availability

Data sharing not applicable to this article as no datasets were generated or analyzed during the current study.

## Abbreviations

ACC: American College of Cardiology
ACE: Angiotensin converting enzyme
ACPC: Adult Congenital and Pediatric Cardiology
AHA: American Heart Association
CAD: Coronary Artery Disease
COG: Children’s Oncology Group
CV: Cardiovascular
ECG: Electrocardiogram
HF: Heart failure
HL: Hodgkin lymphoma
LV: Left Ventricle
MR: Magnetic resonance
MRB: Mineralocorticoid receptor blocker
MUGA: Multi-gated acquisition scan
VAD: Ventricular assist device
