# Supplementary material for: The landscape of cardiovascular care in pediatric cancer patients and survivors: a survey by the ACC Pediatric Cardio-Oncology Work Group
Source: Cardiooncology. 2019 Oct 23;5:16. doi: 10.1186/s40959-019-0051-8 (PMC7048086; doi:10.1186/s40959-019-0051-8)
Supplement: Supplementary file 1 — Additional file 1. Questions for Pediatric Cardio-Oncology Practice Survey. [file 40959_2019_51_MOESM1_ESM.pdf]

Questions for *Pediatric Cardio-oncology Practice Survey*:

Demographic

1. What is your job designation?
    a. Physician
    b. Advanced Practice Nurse
    c. Physician Assistant
    d. Nurse
    e. Other

2. Which of the following best applies to your practice environment?
    a. Academically affiliated
    b. Owned by a corporation
    c. Privately owned
    d. Owned by a hospital or large insurer
    e. None of the above apply

3. Who is responsible for managing cardio-oncology* issues (inpatient and/or outpatient) at your institution?
    a. Dedicated cardio-oncology service
    b. Pediatric cardiologist(s)
    c. Pediatric oncologist(s)
    d. Shared between Pediatric Cardiology and Oncology
    e. Adult Cardio-Oncology specialist
    f. Other (<u>write in</u>)
    g. Unknown

*This may include, but is not limited to, patients before, during, or after exposure to chemotherapy and/or radiation therapy with: symptomatic heart failure, asymptomatic ventricular dysfunction, pericardial effusion, ECG changes, arrhythmia, chest pain.

Patient Population

4. Which of the following patient populations are referred for assessment by a Cardiologist at your institution?
    a. All anthracycline exposure
    b. All chemotherapy exposure, regardless of type
    c. All radiation exposure, regardless of chemotherapy
    d. Abnormal testing (ECG, echocardiogram, etc.) in patients after cancer treatment
    e. None of the above apply
    f. Unknown

5. Which of the following patients are seen for Cardio-Oncology in your institution (any stage of therapy)?
    a. Pediatric only
    b. Pediatric and adult
    c. Does not apply
    d. Unknown

Surveillance Practices

6. Does practice at your institution include screening of patients <u>prior to or during cancer treatment</u>?
    a. Yes, all patients prior to therapy
    b. Yes, all patients once therapy is started
    c. Yes, but only if included in treatment protocol
    d. No
    e. Unknown

7. If the answer to #6 above was "a, "b," or "c," which of the following are *regularly* used? Please check all that apply.
    a. ECG
    b. Extended arrhythmia monitoring (Holter, etc.)
    c. Echocardiogram, standard (includes shortening fraction and/or ejection fraction)
    d. Echocardiogram, advanced (includes diastolic parameters, strain imaging, stress)
    e. Cardiac MRI
    f. Radionuclide imaging (MUGA)
    g. Cardiopulmonary exercise testing
    h. Serum biomarkers
    i. Evaluation by a cardiologist
    j. Other (<u>write in</u>)
    k. None

8. If patients are followed regularly <u>prior to or during cancer treatment</u>, how is the evaluation interval determined?
    a. Children's Oncology Group (COG) recommendation for screening in survivors
    b. Chemotherapy protocol, when there are surveillance guidelines included
    c. Other literature
    d. Similar to other "at risk" or cardiomyopathy populations
    e. Unknown

9. At your institution, is a cardiologist involved in decisions to use cardioprotective agents (e.g. dexrazoxane) or to alter therapy for cardiac-specific concerns?
    a. Yes, all patients
    b. Yes, some patients as determined by Oncology
    c. No
    d. Unknown

10. Does your institution have a clinic for patients <u>after cancer treatment</u> (“survivor” clinic)?
    e. Yes, with Cardiology embedded
    f. Yes, without Cardiology embedded
    g. No
    h. Unknown

11. Which of the following are used in assessing patients <u>after cancer treatment</u> (“survivors”)? Please check all that apply.
    a. ECG
    b. Extended arrhythmia monitoring (Holter, etc.)
    c. Echocardiogram, standard (includes shortening fraction and/or ejection fraction)
    d. Echocardiogram, advanced (includes diastolic parameters, strain imaging, stress)
    e. Cardiac MRI
    f. Radionuclide imaging (MUGA)
    g. Cardiopulmonary exercise testing
    h. Evaluation by a Cardiologist
    i. Other
    j. None
    k. Unknown

12. At your institution, when is a patient <u>after cancer treatment</u> (“survivors”) seen by a cardiologist?
    a. At all COG-recommended surveillance intervals
    b. Only when an abnormality is found on COG-recommended testing
    c. Only with symptomatic heart failure
    d. Pregnancy
    e. Other
    f. None of the above apply
    g. Unknown

13. At your institution, are exercise recommendations provided for patients <u>after cancer treatment</u> (“survivors”)?
    a. Aerobic exercise, but not isometric/weightlifting, is restricted
    b. Isometric/weightlifting, but not aerobic exercise, is restricted
    c. All exercise is restricted
    d. Exercise prescription is tailored to specific patient needs and cardiac health
    e. There is no discussion of exercise recommendations or limitations
    f. None of the above apply
    g. Unknown

14. Considering cardiovascular health in patients after cancer treatment (“survivors”), which of the following issues are addressed by a cardiologist? Please check all that apply.
    a. Ventricular function
    b. Blood pressure
    c. Lipids
    d. Diet, exercise, weight loss
    e. Other cardiovascular risk factors (obesity, smoking, etc.)
    f. Coronary artery health
    g. None
    h. Unknown

Management Practices

15. Regarding the decision to treat ventricular systolic dysfunction in patients before, during, or after cancer treatment, which of the following parameters are used to determine when to start therapy?
    a. Left ventricular shortening fraction (LVSF)
    b. Left ventricular ejection fraction (LVEF)
    c. Strain value regardless of LVSF or LVEF
    d. A, B, or C if any is abnormal
    e. Other
    f. Unknown

16. Regarding patients with ventricular dysfunction before, during, or after cancer treatment, which of the following medications do you prescribe? Check all that apply.
    a. Angiotensin converting enzyme inhibitor (or angiotensin receptor blocker)
    b. Beta blocker
    c. Mineralocorticoid receptor blocker
    d. Statin
    e. Diuretics
    f. Other
    g. Unknown

17. Does your institution offer heart transplantation?
    a. Yes, including to patients after cancer treatment (see question #18)
    b. Yes, excluding patients after cancer treatment
    c. No

18. If the answer to #17 above is “A” what is the minimum time off therapy or in remission to qualify for heart transplantation? Please pick the option that is most appropriate.
    a. > 1 year
    b. >2 years
    c. >5 years
    d. Does not apply
    e. Unknown

19. Does your institution implant ventricular assist devices (VAD)?
    a. Yes, including to patients after cancer treatment (see question #20)
    b. Yes, excluding patients after cancer treatment
    c. No

20. If the answer to #19 above is “A” what is the minimum time off therapy or in remission to qualify for VAD? Please pick the option that is most appropriate.
    a. > 1 year
    b. >2 years
    c. >5 years
    d. No minimum
    e. Does not apply
    f. Unknown
